# Experiencing Lifetime Domestic Violence: Associations with Mental Health and Stress among Pregnant Women in Rural Bangladesh: The MINIMat Randomized Trial

**DOI:** 10.1371/journal.pone.0168103

**Published:** 2016-12-19

**Authors:** Shirin Ziaei, Amy Lynn Frith, Eva-Charlotte Ekström, Ruchira Tabassum Naved

**Affiliations:** 1 Department of Women´s and Children´s Health, Uppsala University, Uppsala, Sweden; 2 School of Health Sciences and Human Performance, Ithaca College, Ithaca, New York, United States of America; 3 International Centre for Diarrhoeal Disease Research (ICDDR, B), Dhaka, Bangladesh; University of Oxford, UNITED KINGDOM

## Abstract

**Background:**

Experience of domestic violence has negative mental health consequences for women. The association of cumulative and specific forms of domestic violence, particularly emotional violence and controlling behavior, with common mental disorders and stress has rarely been studied in pregnant women. The aim of this study is to evaluate associations of specific and multiple forms of lifetime domestic violence and controlling behavior with distress and cortisol level during pregnancy in rural Bangladeshi women.

**Methods and findings:**

In this observational sub-study of larger MINIMat trial, 3504 pregnant women were interviewed using a shortened Conflict Tactic Scale about their lifetime experience of domestic violence including physical, sexual, emotional domestic violence and controlling behavior. Women’s levels of emotional distress were assessed using the self-reported questionnaire (SRQ-20) developed by WHO, and levels of morning salivary cortisol were measured in a subsample (n = 1300) of women during week 28–32 of pregnancy. Regression analyses were used to estimate the associations of lifetime physical, sexual, emotional domestic violence and controlling behavior with levels of distress and cortisol during pregnancy. The prevalence of lifetime domestic violence was 57% and emotional distress was 35% in these pregnant women. All forms of domestic violence were associated with higher levels of emotional distress. Women who experienced either emotional violence or controlling behavior had the highest levels of emotional distress. There was a dose-response relationship between cumulative number of the different forms of domestic violence and women’s levels of emotional distress. There was no association between women’s experience of domestic violence and level of morning salivary cortisol.

**Conclusion:**

Including emotional violence and controlling behavior as major types of violence in future research and health interventions is warranted. Furthermore, the extent of the negative impacts of domestic violence on pregnant women, multiple forms of violence and their cumulative effects need to be investigated.

## Introduction

Gender-based domestic violence (DV) has been defined as any act of physical, sexual, emotional abuse and controlling behavior against a woman by an intimate partner or a family member. In most cases, the intention is inflicting harm or exercising power over a women and the perpetrator is her intimate partner [1,2]. Worldwide almost one-third of the women have experienced physical and/or sexual intimate partner violence (IPV) in their lives (2).

There is growing evidence that the experience of DV is associated with poor mental health and poor birth outcomes among women of reproductive years [3,4,5,6]. Poor mental health may increase onset and severity of physical health problems and poor birth outcomes through the reduction of the women’s ability to self-care and poor adherence to perinatal and preventive care [7]. Furthermore, experience of psychological distress during pregnancy may negatively affect fetal growth [8] and alter offspring’s immune and metabolic functions, thereby making them more susceptible to diseases later in life [9,10].

Alteration of the hypothalamic-pituitary-adrenocortical (HPA) system and normal regulation of cortisol [11] has been suggested as a critical mediator of stress that influences women’s health and birth outcomes. While the normal diurnal pattern of cortisol includes a morning peak, and a late evening nadir [12,13], the HPA system adapts to chronic stress by increasing morning cortisol [12], and to trauma by both lowering morning and increasing evening cortisol [13]. The limited research of DV and cortisol among non-pregnant and pregnant women is contradictory, however, as the experience of DV has been associated with higher levels of morning cortisol [14], or no change in levels of morning cortisol with higher levels of evening cortisol [12,15]. While effects of these adaptations are not fully known, an altered level of cortisol can disturb women’s neural, neuroendocrine and immune functions, making them more susceptible to metabolic diseases, immune disorders [16,17], and poor birth outcomes [18,19].

Despite the fact that the association of DV with common mental disorders has been studied extensively among women, some important questions remain. Several studies have suggested emotional abuse and controlling behaviors as distinct forms of violence that might co-occur [20,21]. Few studies, however, have examined the specific associations of each of these forms of violence and their cumulative influence along with physical and sexual violence on distress and cortisol level, especially among pregnant women in low-income settings.

Bangladesh is a country with high prevalence of DV. Despite the fact that the country has a legal framework for addressing domestic violence, around 53% of ever married Bangladeshi women reported a lifetime experience of physical and/or sexual violence by their partner [22]. Besides, the patriarchal structure of this society allows men’s controlling behavior to be normal and acceptable in marriage [23]. While only a few studies have been conducted on the extent of mental health disorders in Bangladeshi women [24,25], these studies suggest that they are highly prevalent. As noted previously, physical and sexual DV can contribute to poor mental health and stress among women, but little is known about the relationships of emotional violence and controlling behaviors with these outcomes. Thus, the present study aims to evaluate associations between specific and multiple forms of lifetime DV with experience of emotional distress during pregnancy among women who live in rural Bangladesh. The second aim of the study is to evaluate association between specific and multiple forms of lifetime DV and cortisol level in this sample.

## Materials and Methods

### Subjects and study design

The study was part of a larger study registered as Maternal and infant nutrition intervention, Matlab (MINIMat-Trial reg#ISRCTN16581394). MINIMat is a population-based food and micronutrient supplementation trial of pregnant women. The study design and procedures have been described in detail previously [26]. In brief, the study site was in Matlab, a rural sub-district in Bangladesh where International Centre for Diarrhoeal Disease Research, Bangladesh (ICDDR, B) has been conducting an ongoing health and demographic surveillance system since 1966. During November 2001-October 2003 all women who were identified and confirmed pregnant (< 14 weeks gestation) in the study area were encouraged to visit ICDDR, B clinics for further assessments. Enrolled pregnant women (n = 4436) were randomly allocated into 2 types of foods and 3 types of micronutrients supplementation in a 2 by 3 factorial design. Each participant was also randomly assigned to receive either standard ongoing monthly health counseling or health and exclusive breastfeeding counseling, started at third trimester. Women were followed-up monthly at home, and visited the clinics at weeks 14, 19 and 30 of their pregnancy.

### Data collection and measurements

Through a household/clinic visit at around week 8 of pregnancy, maternal characteristics including BMI, age, socioeconomic status (SES), educational level and household characteristics were recorded by using a pre-coded questionnaire.

#### Lifetime experience of DV

During a woman’s clinic visit at week 30, a team of trained paramedics interviewed her regarding her experience of DV by her intimate partner or a family member. A shortened and modified version of Conflict Tactic Scale (CTS) [27] was used to assess each woman’s experience of lifetime DV. The questionnaire has been used in the WHO multi-country study on Women’s Health and Domestic Violence against Women [28], and was adapted for implementation in Bangladesh based on qualitative formative research. For the current study, the items were modified for capturing DV. Further, only one question on sexual violence was asked using wording to capture different sexual acts of violence. The translated version of the questionnaire was pre-tested and the modified version was back-translated, pilot tested, and modified before use. The respondents were asked about their experience of specific acts of physical, sexual, emotional DV as well as controlling behavior by their intimate partner or a family member. Physical violence refers to intentional use of physical force with the potential for causing death, injury or harm. Sexual violence refers to any sort of harmful or unwanted sexual behaviour that is imposed on someone. Emotional violence includes a range of behaviours that causes emotional harm or trauma and controlling behaviour includes a range of behaviours that are imposed on someone to control him/her. The questions used to document DV are listed in Table 1. In general, good internal consistency was observed among each specific measurement with Cronbach’s alpha for physical DV (0.75), emotional DV (0.73) and controlling behavior (0.68). Cronbach’s alpha for sexual DV was not measurable since there was only one question regarding sexual DV. Using factor analysis, we found four distinct groups and each item loaded on its expected factor.

**Table 1:** Questions used to document lifetime physical, sexual, emotional DV and controlling behavior in the MINIMat study.

**Physical DV**
***Moderate***
Has your husband or anyone else from your family ever slapped you or threw something at you that could hurt you?
Has your husband or anyone else from your family ever pushed you or shoved you?
***Severe***
Has your husband or anyone else from your family ever hit you with his fist or with something else that could hurt you?
Has your husband or anyone else from your family ever kicked you, dragged you or beat you up?
Has your husband or anyone else from your family ever choked or burnt you on purpose?
Has your husband or anyone else from your family ever used a knife, gun or other weapon against you?
**Sexual DV**
Has your husband or anyone else from your family ever forced you to have sex or perform any sexual act when you did not want to?
**Emotional DV**
Has your husband or anyone else from your family ever insulted you or made you feel bad about yourself?
Has your husband or anyone else from your family ever belittled or humiliated you in front of other people?
Has your husband or anyone else from your family ever done things to scare or intimidate you on purpose?
Has your husband or anyone else from your family ever threatened to hurt you or someone you care about?
**Controlling behavior**
Has your husband or anyone else from your family ever tried to restrict your contact with your family of birth?
Has your husband or anyone else from your family ever tried to restrict your contact with your friends and neighbors?
Has your husband or anyone else from your family ever ignored you and treated you indifferently?
Has your husband or anyone else from your family ever got angry if you spoke with another man?
Has your husband or anyone else from your family often been suspicious that you were unfaithful?

#### Emotional distress

Each participant’s level of emotional distress were assessed using self-reported questionnaire (SRQ-20), developed by WHO to screen for emotional distress, especially in non-Western cultures [29]. The reliability and validity of the SRQ-20 has been established in several countries [29,30,31]. The questionnaire was pilot-tested in 30 pregnant women within the community and a separate cognitive interview was conducted to evaluate women’s understanding of the questions. The Bengali version of the study protocol was translated and back-translated by bilingual research assistants [32]. The Cronbach’s alpha for the questionnaire was 0.93 in our sample, indicating a good internal consistency. The questionnaire consists of 20 yes/no questions, with the maximum score of 20 and a higher score reflecting a higher level of emotional distress. Each participant was asked whether she had experienced symptoms during the last 4 weeks that are associated with emotional distress, such as headache, tiredness, crying and suicidal thoughts.

#### Salivary cortisol

Salivary cortisol was measured in a subsample of women during week 28–32 of pregnancy. Samples were collected from June 2003 until March 2004 resulting in a total of 1300. Each participant was visited in her home by a field worker between 7–8 am (approximately 30 min to 1 h post awakening) for the collection of one salivary sample. The timing of cortisol collection in this population has been validated as a representative measure of morning cortisol [33]. Each participants was given a cylindrical cotton swab, chewed on it for 30–45 s or until it was fully saturated, and placed it in a test tube with cap. Collected samples were frozen, and stored at -20°C on the same day. Concentration of cortisol was further analyzed by using solid phase I radioimmunoassay by the laboratory of Dirk Hellhammer, University of Trier, Trier, Germany.

### Data coding

Independent variables: A wealth index was created based on land ownership, dwelling characteristics and household ownership of durable (*e*.*g*., bed, radio, TV, electric fan) and other goods to assess SES [34] and further divided into quintiles. A participant’s level of education categorized into none, 1–5 years, and 6 years and above. Based on each woman’s answers to the DV questionnaire the following binary variables (yes/no) were developed: the experience of any form of violence including moderate physical DV, severe physical DV, physical (either moderate or severe) DV, sexual DV, emotional DV, and controlling behavior. Lifetime experience of DV (yes/no) was defined based on a woman’s experience of one or more acts of violence. The variable “cumulative number of different forms of DV” (range 0–4) was created by summing up different forms of DV (*i*.*e*., physical, sexual, emotional and controlling behavior) that an individual experienced.

Outcome variables: A Woman’s experience of emotional distress (yes/no) was defined based on cut-off score that has been used in previous studies in Bangladesh [35], and each woman was categorized as being emotionally distressed if her SRQ-20 score was equal to or greater than 7.

### Ethics

The study followed WHO ethical and safety guidelines on conduct of domestic violence research. Informed written consent was obtained from participating women at enrollment. Female paramedics received training on data collection regarding violence against women and emotional distress, and they conducted the interviews in private upon receipt of informed consent. As described in a previous paper by Naved et al. [36] a woman who reported physical or sexual violence or suicidal ideation or attempt was offered mental health counselling. Strict confidentiality of each participant’s information was maintained while handling the data. The study was reviewed and approved by the ethical review committee of ICDDR, B.

### Statistical analyses

Descriptive characteristics of the women are presented as frequencies and percentages for categorical variables and mean and standard deviation (SDs) for continuous variables. Normality of cortisol data was evaluated and established by visual examination of Q-Q plots and histograms. We used *chi-square* tests to compare proportions and *t* tests / analysis of variance (ANOVA) followed by Bonferroni *post hoc* tests to compare means and evaluate factors associated with a woman’s emotional distress and level of cortisol. Finally we used logistic regressions for binary (emotional distress) and general linear models for continuous (cortisol) outcomes to estimate associations with explanatory variables. The results of this study are presented in two types of models: unadjusted models and models adjusted for potential confounders. Based on the previous research on the determinants of DV [37,38] and mental health [39] in Bangladesh, as well as, factors associated with maternal level of cortisol [40], the following factors were selected as potential confounders in this study: maternal age, education, socio-economic status (SES), BMI, parity, child sex and prenatal food and micronutrient supplementation groups to which the women were allocated. All models were adjusted for women’s age, education (none, 1–5 years, and 6 years and above) and SES (quintiles). Models evaluating level of cortisol were adjusted additionally for women’s BMI as it was associated with DV and cortisol in this study. Child sex and food and micronutrient supplementations groups were removed from final analyses as they did not alter the model parameters. Parity was excluded from final analyses since it was highly correlated with women’s age (r = 0.78, p<0.01). Statistical analyses were performed with statistical software packages IBM SPSS Statistics version 20 and openEpi version 3.03a.

## Results

**Fig 1:**
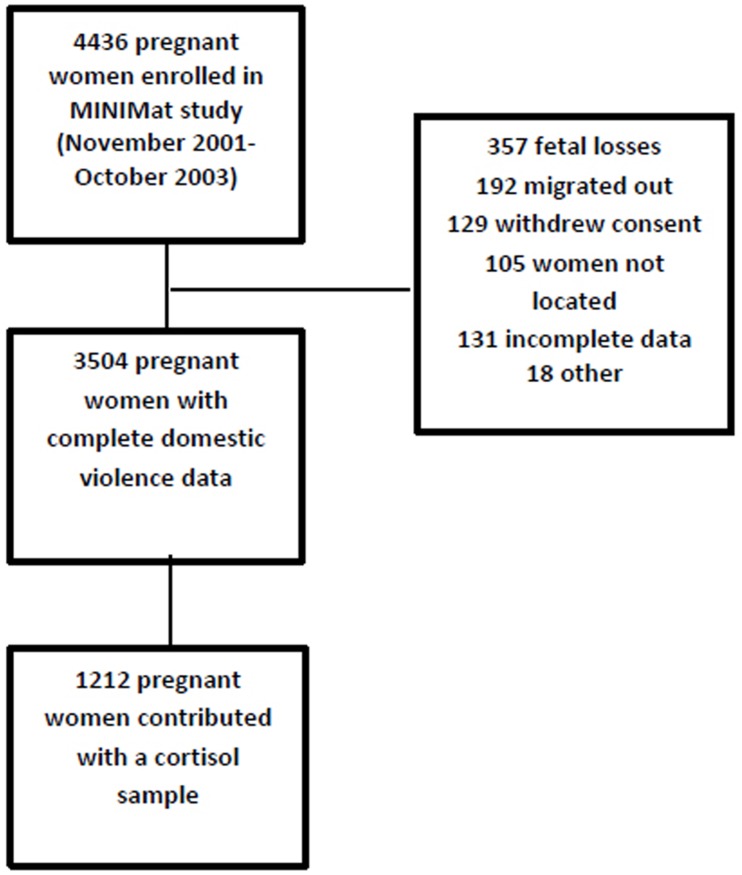
Flowchart of participating women.

Out of 4436 enrolled pregnant women 3504 were included in this study (Fig 1). The main reasons for lost to follow up were fetal loss, out migration and withdrawal of consent especially during Ramadan. Women who had incomplete data on exposure to DV were also excluded from the study. There were no significant differences between demographic and anthropometric characteristics of final sample and those who were lost to follow up or excluded from the analyses (data not shown). Descriptive characteristics of study sample are presented in Table 2. The average age of women was 27 years and their mean BMI was 20. One-third of the women had no formal education and around one-third were expecting their first child. There were no significant differences in demographic and anthropometric characteristics of the total sample and the subsample of women (n = 1212) in which levels of cortisol were measured.

**Table 2:** Descriptive characteristics of participating pregnant women.

Variable	n (%) or mean±SD ^a^
Age in years	26.28±5.87
BMI	20.40±2.52
Education	
• none	1116 (31.8)
• 1–5 years	757 (21.6)
• ≥ 6 years	1631 (46.5)
Parity	
• 0	1162 (33.2)
• 1	997 (28.5)
• 2	719 (20.5)
• 3	365 (10.4)
• ≥ 4	256 (7.3)
Experience of DV	
• Lifetime DV	2010 (57.4)
• Any physical DV	759 (21.7)
∘ Moderate physical DV	739 (21.1)
∘ Severe physical DV	276 (7.9)
• Any sexual DV	846 (24.1)
• Any emotional DV	962 (27.5)
• Any controlling behavior	1288 (36.8)
Number of forms of DV experiences	
• None	1494 (42.6)
• 1	913 (26.1)
• 2	541 (15.4)
• 3	364 (10.4)
• 4	192 (5.5)
Emotional distress (SRQ ≥ 7)	1239 (35.4)
Salivary cortisol (nmol L^−1^) ^b^	9.88±3.53

DV, Domestic Violence; SRQ, Self-Reported Questionnaire

^a^ n = 3504, total sample size varies due to missing values.

^b^ n = 1212

More than 57% of the women experienced at least one form of DV in their life. The most common form of DV was controlling behavior (36.8%) followed by emotional DV (27.5%). More than 31% of women experienced more than one form of DV and around 35% showed emotional distress with SRQ scores ≥ 7 (Table 2).

Distribution of different forms of violence and prevalence of emotional distress and mean levels of salivary cortisol are presented in Table 3. Prevalence of emotional distress was 46% among the women who reported any lifetime DV. All forms of DV were associated with emotional distress. Out of all forms of DV, the highest proportions of women with emotional distress were among those who experienced severe physical DV (62.9%) or emotional DV (57.8%). As the cumulative number of different forms of violence increased, the proportion of women with emotional distress increased, such that 70.3% of women who had experienced all forms of violence were emotionally distressed. Salivary cortisol level was not associated with the experience of lifetime DV (yes/no), any form of DV, or the cumulative number of different forms of violence.

**Table 3:** Descriptive statistics of women’s experience of lifetime DV and their emotional distress and level of salivary cortisol.

		Emotional distress (SRQ ≥ 7)					Salivary Cortisol (nmol L^−1^)		
		n	%	95% CI	P-value ^a^	n	Mean	SD	P-value ^b^
Lifetime DV	Yes	923/2005	46.0	43.9–48.2	<0.01	698	9.84	3.49	0.57
	No	316/1492	21.2	19.2–23.3		514	9.95	3.60	
Any physical DV	Yes	399/757	52.7	49.2–56.2	<0.01	282	9.82	3.50	0.75
	No	840/2740	30.7	29.0–32.4		930	9.90	3.55	
• Moderate physical DV	Yes	388/737	52.6	49.0–56.2	<0.01	277	9.83	3.53	0.78
	No	851/2759	30.8	29.2–32.6		935	9.90	3.54	
• Severe physical DV	Yes	173/275	62.9	57.6–68.4	<0.01	112	9.64	3.06	0.45
	No	1066/3222	33.1	31.5–34.7		1100	9.91	3.58	
Any sexual DV	Yes	430/844	50.9	47.6–54.3	<0.01	276	10.04	3.30	0.41
	No	809/2653	30.5	28.7–32.2		936	9.84	3.60	
Any emotional DV	Yes	554/958	57.8	54.7–60.9	<0.01	375	9.75	3.42	0.38
	No	685/2539	27.0	25.3–28.7		837	9.94	3.58	
Any controlling behavior	Yes	640/1284	49.8	47.1–52.6	<0.01	446	9.74	3.52	0.28
	No	599/2213	27.1	25.3–29.0		766	9.97	3.54	
Cumulative number of different forms of DV									
• None		316/1492	21.2	19.2–23.3	<0.01	514	9.95	3.60	0.86
• 1		306/913	33.5	30.5–36.6		305	9.89	3.50	
• 2		269/538	50.0	45.8–54.2		190	9.69	3.61	
• 3		213/362	58.8	53.7–63.8		118	10.04	3.51	
• 4		135/192	70.3	63.5–76.3		85	9.67	3.16	

DV, Domestic Violence; SRQ, Self-Reported Questionnaire

^a^ p values were calculated using chi-square tests

^b^ p values were calculated using *t* tests / analysis of variance (ANOVA)

Controlling for age, education and SES, women who experienced lifetime DV were more likely to be emotionally distressed (OR _adj_3.18; 95% CI 2.72, 3.70) than non-abused women. All forms of violence were significantly associated with emotional distress in this sample, and the odds of being distressed was highest among the women who experienced emotional DV (OR _adj_3.68; 95% CI 3.14, 4.30). Women who experienced more forms of DV were more likely to be distressed and the odds of being distressed was highest among women who experienced all forms of DV (OR _adj_8.79; 95% CI 6.26, 12.34). There was no association between lifetime experience of DV and a woman’s level of salivary cortisol. Similarly, no associations were observed between forms and the cumulative number of different forms of DV with levels of salivary cortisol (Table 4).

**Table 4:** Association of women’s emotional distress and level of salivary cortisol with experience of lifetime DV.

	Emotional distress (SRQ ≥ 7)		Cortisol (nmol L^−1^)	
	Unadjusted OR ^a^	Adjusted OR ^b^	Unadjusted ß ^c^	Adjusted ß ^d^
No DV	Ref	Ref	Ref	Ref
Lifetime DV	3.18 (2.73–3.70) ^e^	3.18 (2.72–3.70)^e^	-0.12 (-0.52,0.29)	-0.15 (-0.55,0.25)
Any physical DV	2.52 (2.14–2.97) ^e^	2.45 (2.06–2.91) ^e^	-0.08 (-0.55,0.39)	-0.11 (-0.60, 0.38)
• Moderate physical DV	2.49 (2.11–2.94) ^e^	2.41 (2.03–2.87) ^e^	-0.07 (-0.54,0.41)	-0.10 (-0.59, 0.39)
• Severe physical DV	3.43 (2.66, 4.43) ^e^	3.25 (2.50,4.22) ^e^	-0.27 (-0.96,0.42)	-0.29 (-1.00, 0.41)
Any sexual DV	2.37 (2.02–2.77) ^e^	2.32 (1.98–2.73) ^e^	0.20 (-0.28, 0.68)	0.21 (-0.27, 0.68)
Any emotional DV	3.71 (3.18–4.34) ^e^	3.68 (3.14–4.30) ^e^	-0.19 (-062, 0.24)	-0.10 (-0.54, 0.34)
Any controlling behavior	2.68 (2.32–3.09) ^e^	2.74 (2.37–3.17) ^e^	-0.23 (-064, 0.18)	-0.24 (-0.65, 0.18)
Cumulative number of different forms of DV				
• None	Ref	Ref	Ref	Ref
• 1	1.88 (1.56–2.26) ^e^	1.90 (1.58–2.30) ^e^	-0.06 (-0.56, 0.44)	-0.15 (-0.65,0.35)
• 2	3.72 (3.02–4.59) ^e^	3.80 (3.08–4.70) ^e^	-0.26 (-0.85, 0.33)	-0.24 (-0.83, 0.35)
• 3	5.32 (4.17–6.79) ^e^	5.31 (4.15–6.80) ^e^	0.09 (-0.62, 0.80)	0.06 (-0.66, 0.77)
• 4	8.81 (6.31–12.30) ^e^	8.79 (6.26–12.34) ^e^	-0.28 (-1.09, 0.53)	-0.21 (-1.04, 0.61)

DV, Domestic Violence; Ref, Reference category

^a^ Unadjusted OR for emotional distress among women with experience of different forms of DV compared to the women with no experience of DV using logistic regression.

^b^ Adjusted OR for emotional distress among women with experience of different forms of DV compared to the women with no experience of DV using logistic regression. Models adjusted for women’s age, education and SES.

^c^ Unadjusted ß for mean level of cortisol among women with experience of different forms of DV compared to the women with no experience of DV using general linear models.

^d^ Adjusted ß for mean level of cortisol among women with experience of different forms of DV compared to the women with no experience of DV using general linear models. Models adjusted for women’s BMI, age, education and SES.

^e^ p value<0.01

## Discussion

Domestic violence and emotional distress are commonly experienced by pregnant women in this area of Bangladesh. Women who experienced any lifetime DV were more likely to experience emotional distress. All forms of DV were significantly associated with higher level of emotional distress, and women who experienced emotional violence and controlling behavior experienced the highest levels of emotional distress. A Woman’s experience of lifetime DV was not associated with level of morning salivary cortisol.

Our finding that experience of DV was associated with emotional distress is consistent with previous findings. In the WHO multi-country study on women’s health and domestic violence, women who experience IPV report higher level of emotional distress than those who do not in all study sites [4]. Similarly, in postpartum Bangladeshi women [41,42] and South Asian women of reproductive age [43], higher levels of emotional distress and common mental disorders occur among those who experience IPV.

Notably, we found the highest prevalence of emotional distress among women who experienced emotional violence and controlling behaviors as compared to sexual and any physical DV. If women had experienced any emotional violence, they had the highest odds of being distressed. Consistent with these findings, researchers from Paraguay [44] and Brazil [45] reported that compared to other forms of violence, the experience of emotional violence is most strongly associated with higher emotional distress among women of reproductive age. In Bangladesh, strong associations between emotional violence and suicidal ideation were reported among women both in rural and urban areas [46]. Similarly, in qualitative studies, women perceived that the most difficult violence with which to cope was emotional violence [47,48]. Experience of DV, and in particular emotional violence, may attack women’s sense of self, destroy their self-esteem and make them feel they are unworthy and unlovable [49]. Furthermore, associations between controlling behavior and mental health disorders have also been reported from studies in Brazil [45] and Ethiopia [50] among women of reproductive age. Experiencing controlling behavior not only may cause women to feel powerless and hopeless [51], but also might limit women’s social support and reduce the protective effects of such support on negative impacts of violence on their mental health [52].

Around 31% of women in our study experienced more than one form of DV in their life, and there was a dose-response relationship between number of forms of DV and women’s emotional distress. Despite that increased risk of mental health symptoms in the general population of women who experienced multiple forms of violence has been reported from previous studies [45,53]; most studies have focused on physical and sexual violence. In this study, we examined the cumulative effect of physical and sexual violence with the inclusion of emotional violence and controlling behaviors. Coexistence of different forms of violence might reflect more intense DV, which has a more detrimental effect on women’s mental health than one form alone [54].

No association between women’s experience of DV and their morning level of cortisol was observed in our study. This result is consistent with a previous study of pregnant women (18–39 weeks of gestation) experiencing IPV [19]. In contrast, non-pregnant women who experienced IPV had lower morning cortisol than those who did not experience IPV [55]. During pregnancy, maternal level of salivary cortisol starts to rise steadily at around week 25 of gestation and the morning peak concentration is double that of non-pregnant women [56]. This high level of morning cortisol may mask the response to some stressors during late pregnancy [57]. Consistent with these findings, levels of maternal morning cortisol were not associated with psychological stress in late pregnancy, but were in earlier pregnancy [58]. Although, morning cortisol has been suggested and used as a measure to understand the relationship of stressors and outcomes during pregnancy [59], results from the current and some previous studies suggest that evening levels of cortisol may be a better measure of the physiological response to IPV among women during late pregnancy [15,19].

The strengths of this study are a large sample size and the inclusion of different forms of DV in the analysis. Considering the observational design of the study, causal inference cannot be drawn. However, since we studied associations between lifetime experience of DV and recent symptoms of emotional distress among women, our findings suggest temporal association between DV and emotional distress. Although the study was embedded into an interventional trial, we carefully examined the effects of food and micronutrient interventions in the models. In addition, given the health and exclusive breastfeeding counselling began just as the DV interviews and cortisol collection started, they would not influence the account of DV, distress or cortisol. Measuring recent experience of DV rather than lifetime could be hypothesized to provide a better indicator of DV in relation with emotional distress. However, measuring lifetime experience of DV may be more important considering the long-term effects of DV on women’s mental and physical health [4]. Although women’s lifetime experience of DV, in particular emotional DV and controlling behaviors, is subject to recall bias, we attempted to reduce such bias by using a standardized instrument with behaviorally-specific questions. Furthermore, the addition of measuring evening cortisol sample may have provided better information on the stress response to DV. Finally, although we adjusted the analyses for the potential confounders, we could not adjust for potential confounders such as women’s personality, coping strategies and other life situations that were not measured.

The results of current study add to existing literature further outlining the detrimental effects of DV on women’s mental health. Although mental health care is essential for a victim of DV, such services are rarely available in low-income countries such as Bangladesh. Setting up services alone, however, may not benefit abused women as the social barriers to accessing care and services need to be addressed. Out of the several forms of violence we measured (i.e. any physical, sexual, emotional DV and controlling behavior), our findings that emotional violence and controlling behavior were associated with the highest level of emotional distress merits attention. Despite the fact that emotional violence and controlling behaviors are the most frequent forms of violence [60], the literature on the impacts of physical and sexual violence on women’s health overshadows the research on these more common forms of violence. The lack of attention on emotional violence and controlling behavior may occur as they are not considered life threatening, and women’s perception of them as violence may differ among cultures. The effects of such violence, however, are as much as or more detrimental to women’s health as physical and sexual violence [61]. Additionally, in several studies the experience of emotional violence and controlling behavior in early marriage are strong predictors of later physical violence [49,62]. These findings highlight the importance of including both emotional violence and controlling behaviors as major types of violence in future research. Furthermore, we found a dose-response relationship between cumulative number of different forms of DV and a woman’s level of emotional distress. Given that DV is generally a complex pattern of different forms of violence, rather than an isolated act [1], it is important that health care professionals and researchers evaluate the impacts of DV on women’s health in the context of multiple forms of violence rather than focusing on a certain form. At the policy level, evidence-based interventions for the prevention of all forms of DV are required in countries such as Bangladesh where DV is widespread.

## Supporting Information

S1 Data MIMIMat data file. Experience of lifetime DV, emotional distress and cortisol level in pregnant women participating in MINIMat trial, the final sample, n = 3504.
(SAV)
